# Walnut Shell Biowaste Valorization via HTC Process for the Removal of Some Emerging Pharmaceutical Pollutants from Aqueous Solutions

**DOI:** 10.3390/ijms231911095

**Published:** 2022-09-21

**Authors:** Anca Andreea Țurcanu, Ecaterina Matei, Maria Râpă, Andra Mihaela Predescu, Andrei-Constantin Berbecaru, George Coman, Cristian Predescu

**Affiliations:** Faculty of Materials Sciences and Engineering, University Politehnica of Bucharest, 060042 Bucharest, Romania

**Keywords:** biowaste, HTC, adsorption, methylene blue, paracetamol, kinetics

## Abstract

This research emphasizes the performance of some eco-friendly carbon materials as hydrochars (HC) obtained by the hydrothermal carbonization (HTC) process applied to walnut shell (WS) biowaste. These materials display promising properties that can be used for environmental applications such as emerging pharmaceutical pollutant retention from water sources. Thus, three hydrochars coded HCWS1, HCWS2, and HCWS3 were obtained using a dynamic autoclave in specific conditions—temperature of 220 °C, autogenous pressure, 1:10 biomass–water weight ratio—and for three different reaction times, 1 h, 6 h, and 12 h. The HCWSs were characterized by means of ATR-FTIR and SEM-EDS analyses and tested as possible adsorbents to assess the removal efficiencies of some emerging pharmaceutical pollutants (paracetamol and methylene blue) by UV–VIS spectrophotometry. Kinetic and adsorption studies were carried out. The best results were obtained for the HCWS3 hydrochar. Further perspectives include an activation step of the hydrochars and their testing on other emerging pharmaceutical pollutants.

## 1. Introduction

Some of the most common problems that the world is facing today include the massive amounts of waste that are being generated daily, the depletion of natural resources, and the pollution of the environment that accompanies population growth, resource exploitation, inadequate means of waste management, and others [1].

In recent years, due to the increasing need to find new, renewable energy alternatives and to safely manage the huge amounts of waste generated annually worldwide, researchers have been required to develop more efficient and reliable technologies to recover and convert biowastes into value-added products [2].

Using various technologies, such as pyrolysis [3], combustion [4], anaerobic digestion [4,5], fermentation [6], etc., biowastes can be valorized into biofuels [7,8], bio-oil [9], valuable chemicals [10,11], and activated carbon-based adsorbents [12,13,14,15,16,17].

Moreover, water pollution has become a real problem in recent years, both for the environment and human health [18,19]. It affects the proper functioning of vegetation, aquatic and animal life, and the human body. It is, therefore, vital to find effective, sustainable, and low-cost ways to combat this problem [20]. The prospect of incorporating of such value-added and environmentally friendly products into procedures that support environmental protection, such as water purification, is provided by the use of biomass in the manufacturing of carbon-based products produced by specific green chemistry methods. In this instance, chemically and mechanically stable activated carbon with high porosity and specific surface area is used in adsorption processes that are crucial in water purification, disinfection, and wastewater treatment, as well as in other applications, including separation and recovery [21].

A green approach to the valorization of biowaste into valuable products with applications in the water protection area is represented by the hydrothermal carbonization (HTC) process, which converts biomass into hydrochar (HC) as a sustainable, eco-friendly carbon material. According to the literature, the HTC process takes place in an autoclave reactor, in the temperature range of 180–260 °C, with autogenous pressures (max. 30 bar) and for different residence times ranging from half an hour to 24 h [22]. Because the HTC operates in an aqueous environment, it is perfectly suited to processing the wet biomass without any drying step. Applying this process, two main products are obtained: (i) a liquid phase that is rich in organic compounds (furans, saccharides, and other valuable chemicals), and (ii) a solid phase that consists of a carbonaceous material defined as a hydrochar (HC) [23,24]. During the HTC process, a small amount of gases is also obtained, consisting of CO, CO_2_, CH_4_, and H_2_ [25].

Some studies in the literature have presented different types of biowastes that have been converted through this process in HCs and further used as adsorbents, including agricultural waste (corn stalks and straws [26], sugarcane bagasse [27], pine needles [28], palm leaves [17]), fruit and vegetable waste (orange peels [29], rotten apples and grape bagasse [30,31], coconut shells [32], avocado seeds [33], rice husk [34]), municipal and animal waste (sewage sludge [35], cardboard and paper sludge, horse manure [34]), and coffee husks [36]. The HCs were used in water decontamination applications for both organic pollutants such as dyes, including methylene blue (MB) [27,37,38], Congo red (CR) [39,40], and crystal violet (CV) [41]; pharmaceuticals, including triclosan (TCS) [34], paracetamol (PRM) [42], diclofenac (DCF), and ibuprofen (IBP) [43]; and inorganic pollutants, such as heavy metals (Pb^2+^ [13,17], Cu^2+^ [35,44], PO_4_^3−^ [45]). Although similar studies have been reported in the literature related to the HC from various agricultural wastes, to our knowledge, there are no reported studies on the use of walnut shells as adsorbents for priority pollutants.

In Romania, a potential source of biowaste that can be valorized through the HTC process is represented by walnut shells (WS), so the waste generated from such a source can be monitored and managed properly in the context of biomass valorization. Given that Romania ranks first in the EU for walnut production, with a quantity of 60,820 tons of walnuts produced in 2021 [46], WS waste is a source of biowaste with great potential for valorization. Because WS are characterized by high calorific value (20 kWh/kg), 3% ash content, and very low moisture content of 8–10%, they are often used as a cheap source of natural biomass fuel [47]. The abundance of walnuts in Romania makes WS a viable renewable and low-cost resource for the preparation of efficient and affordable adsorbents with potential use in water decontamination applications.

The main objective of the study was to reintroduce into the economic circuit some of Romania’s indigenous vegetable waste with potential for valorization by obtaining carbonaceous eco-materials using an inexpensive and non-polluting hydrothermal process. The materials thus obtained were used as adsorbents for the retention of priority pollutants from water sources. In this study, WS were used as a cheap biowaste source in the HTC process to prepare, at 1 h, 6 h, and 12 h residence times, respectively, three different eco-friendly materials (HCWS1, HCWS2, and HCWS3, corresponding to each residence time) that were used as adsorbents in the retention of MB and PRM from synthetic water solutions. The structural, morphological, and compositional properties of the eco-materials were determined by using attenuated total reflectance Fourier transform infrared spectroscopy (ATR-FTIR) and scanning electron microscopy (SEM) coupled with energy-dispersive spectra (EDS). To better understand the adsorption pathways, kinetic and adsorption studies were carried out, these data being some preliminary results for future mechanism adsorption process evaluation.

## 2. Results and Discussion

### 2.1. Characterization of the HCWS Eco-Materials

**Figure 1:**
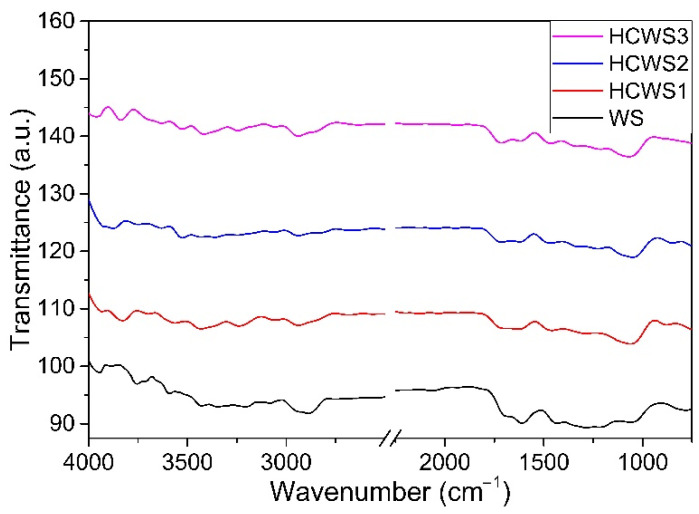
ATR-FT-IR spectra for raw material (WS) and hydrothermal char from walnut shells: HCWS1, HCWS2, and HCWS3, respectively.

The HCWS1, HCWS2, and HCWS3 eco-materials obtained by the HTC process were structurally investigated using ATR-FTIR analysis. The raw material (WS) in powder form, before HTC treatment, was used for comparison purposes and the resulting spectra are shown in Figure 1.

For all the materials investigated, the broad bands identified in the 3000–3700 cm^−1^ region can be attributed to the stretching vibrations of the hydrogen bonds in the alcoholic and phenolic -OH groups present in the cellulose and lignin components [48]. The absorption bands present in the 2800–3000 cm^−1^ range correspond to the -CH stretching vibrations of the -CH_3_- and -CH_2_- groups of lignin in the samples. The intense absorption band at 1730 cm^−1^ corresponding to the stretching vibration of the -C=O bond in the beta position of the acetyl or carboxylic acid groups is observed for WS and HCWS eco-materials, and it is caused by the presence of holocellulose (the carbohydrate fraction of woody materials that is insoluble in water) in these samples [49]. A decrease in the intensity of the absorption bands mentioned above in the spectra of eco-materials obtained by the HTC process could indicate the degradation of the low molecular weight of polysaccharides during hydrothermal treatment. Absorption bands at 1230 cm^−1^, which correspond to axial asymmetric vibrations of the =C-O-C- group, are observed in all samples analyzed, with a higher intensity in the initial walnut shells. This could be related to the higher content of ethers in this WS sample. An intense absorption band at 1040 cm^−1^ is attributed to asymmetric vibrations of the -CH bonds in the aromatic rings of lignin and deformation vibrations of the -CO bonds in the primary alcohols that are found in the hemicellulose present in the WS sample [50]. This band was observed to be less intense in the spectra of the eco-materials prepared with the HTC process, suggesting the decomposition of the hemicellulose components during the hydrothermal treatment of the raw material.

In order to investigate the structural and morphological properties of the WS and HCs, scanning electron microscopy (SEM) coupled with an energy-dispersive spectrometer (EDS) (QUANTA 450 FEG microscope) was used.

**Figure 2:**
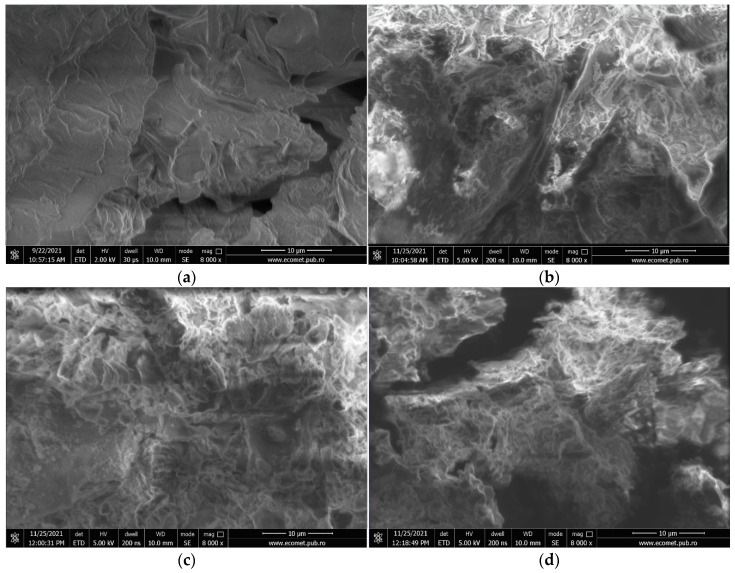
SEM images for WS (**a**) and hydrothermal char from walnut shells: HCWS1 (**b**), HCWS2 (**c**), and HCWS3, respectively (**d**), magnification 8000×, scale 10 μm.

Figure 2a–d show the SEM images of the raw material (WS) and the HCWS1, HCWS2, and HCWS3 eco-materials obtained by the HTC process. The HTC process allows the creation of micro-cracks and pores in the carbonized materials, which will considerably increase their adsorption capacities [51]. The SEM image obtained for raw biomass reveals a surface with a relatively heterogeneous structure, typical for lignocellulosic biomass [52]. The HTC process disturbs the smooth structure of the biomass, leading to the creation of cracks and pores on the HC surface and particle fragmentation (smaller, spherical particles) with an increase in retention time during the process. Samples obtained by HTC at 220 °C showed similar structures to those reported and illustrated in other studies [53,54]. The samples did not present crystals specific to the saccharide structure, indicating that most of the saccharides were hydrolyzed and dissolved during the HTC process [55]. In Figure 2b–d, disorganization of the particles can be seen compared to the lamellar structure of WS (Figure 2a), indicating that almost all of the hemicellulose has been removed. These results signify that the solid residue has been depleted in hemicellulose, which is consistent with the ATR-FTIR analysis. Moreover, the rough surface of the eco-materials could have beneficial effects for pollutant adsorption.

From the EDS analysis, it was observed that, for the WS sample, the composition consisted of C (62.92%), O (36.86%), Si (0.01%), K (0.14%), and Ca (0.09%), while, for the adsorbent eco-materials derived by the HTC process, the composition consisted mainly of C and O. For HCWS1, the composition consists of 67.55% C and 32.45% O; for HCWS2, 72.49% C and 27.51%; and for HCWS3, 73.15% C and 26.85% O. The information obtained suggests that, in the case of the HCWS1, HCWS2, and HCWS3 eco-materials, the water-soluble compounds were dissolved and transferred into the liquid phase obtained in the process, leaving only C and O as the main elements found in the composition of these materials. It is also observed that the C content increases with reaction time.

### 2.2. Removal Efficiency Tests

In order to test the performance of the eco-materials prepared by the HTC process, quantitative analyses of the removal capacity of methylene blue—MB—and paracetamol—PRM—pollutants from aqueous solutions were performed using a UV–VIS spectrophotometer.

The calibration curve for MB was plotted using six MB standards of known concentrations in the range 0.5–5 mg/L, prepared from a stock solution of 100 mg/L. The MB scan showed an adsorption maximum peak at 664 nm. Absorbance analysis for the prepared standards resulted in a data correlation coefficient (R^2^) of 0.9996. The adsorption maximum peak for PRM was recorded at 275 nm and, accordingly, the calibration curve was performed over a concentration range of 5–100 mg/L; the correlation coefficient R^2^ of 0.999 was found.

#### 2.2.1. Effect of Contact Time on Removal Efficiency

**Figure 3:**
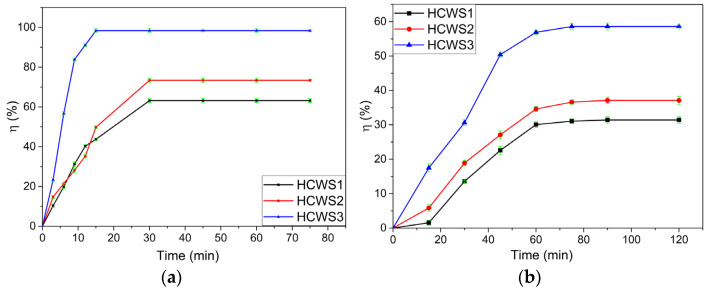
Pollutant retention efficiency for HCWS eco-materials. (**a**) MB, concentration of 5 mg/L; (**b**) PRM, concentration of 50 mg/L.

Contact time is an important factor influencing the adsorption process and has been investigated for MB and PRM pollutants. The retention efficiencies of the HCWS eco-materials are shown in Figure 3a,b.

For the analysis of the MB pollutant, 50 mL of 5 mg/L solution was brought into contact with 0.1 g of adsorbent eco-material. Samples were taken every 3 min during the first 15 min of contact and every 15 min to 75 min thereafter; the taken solutions were analyzed on the UV–VIS spectrometer. A graphical representation of the evolution of the retention efficiency during this time interval is shown in Figure 3a. It was found that all the adsorbent eco-materials showed retention efficiencies above 60%, with a maximum of 63.19% ± 0.65% for HCWS1 and 73.4% ± 0.66% for HCWS2 after a contact time of 30 min, and 98.36% ± 0.96% for HCWS3 after 15 min of contact, respectively.

In the case of the PRM pollutant, a 50 mg/L solution was placed in contact with 0.1 g of adsorbent. Samples were taken every 15 min, but for a contact time of 120 min, since the maximum retention efficiency for PRM was reached at a longer time, as can be seen in Figure 3b. In this case, it was observed that the eco-materials showed retention efficiencies between 30 and 60%—specifically, 31.58% ± 0.69% for HCWS1, 36.96% ± 0.95% for HCWS2, and 59.23% ± 1.04% for HCWS3, respectively, after 90 min of contact.

It was observed that in the case of both pollutants investigated, the better results were obtained when using the HCWS3 eco-material. This can be attributed to the material preparation conditions—specifically, the longer residence time during the HTC process (12 h) for this specific eco-material, which resulted in a larger and more functionalized specific surface area compared to the other materials prepared at lower residence times. This correlates with the morphological and structural characterizations of the eco-materials presented above.

#### 2.2.2. Effect of Adsorbent Amount on Removal Efficiency

**Figure 4:**
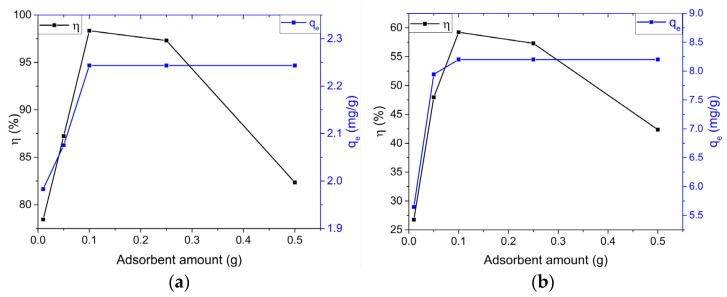
Effect of adsorbent amount on removal efficiency and maximum adsorbed amount by HCWS3; MB (**a**) and PRM (**b**). The black and blue lines represent the removal efficiency (*η*) and the adsorption capacity (*q_e_*), respectively.

For the determination of the optimal amount of adsorbent, proper selection was necessary, as the availability of readily accessible adsorption centers and the active surface area are crucial parameters that significantly affect the removal efficiency of the pollutant [56]. For this study, different amounts of HCWS3 adsorbent in the range of 0.01–0.5 g were used, keeping other parameters (pollutant concentration, solution volume, contact time) constant. The effect of the adsorbent mass used in the experiments on the retention efficiency and adsorption capacity of the pollutants is shown in Figure 4a,b.

According to Figure 4, it is observed that with the increase in the amount of HCWS3 eco-material, the retention efficiency of the investigated pollutants increased up to the amount of 0.1 g adsorbent, where it reached the maximum values for removal efficiencies of both pollutants (98.36% for MB and 59.23% for PRM), followed by an insignificant decrease in efficiency for an amount of 0.25 g.

For the adsorbent amount of 0.5 g, the retention efficiency of the HCWS3 material decreased significantly to values of 83.51% for MB and 42.35% for PRM. The decrease in adsorbent holding capacity with increasing adsorbent mass can be explained as follows: (i) increasing the amount of adsorbent at a constant concentration and volume of pollutant will lead to unsaturation of the adsorption centers during the process, and (ii) it may be due to the interaction of adsorbent particles, such as aggregation resulting from using a higher amount of HCWS3—such aggregation would lead to a decrease in the total surface area of the adsorbent and an increase in the diffusion paths [57].

#### 2.2.3. Effect of the Initial Pollutant Concentration on the Removal Efficiency

**Figure 5:**
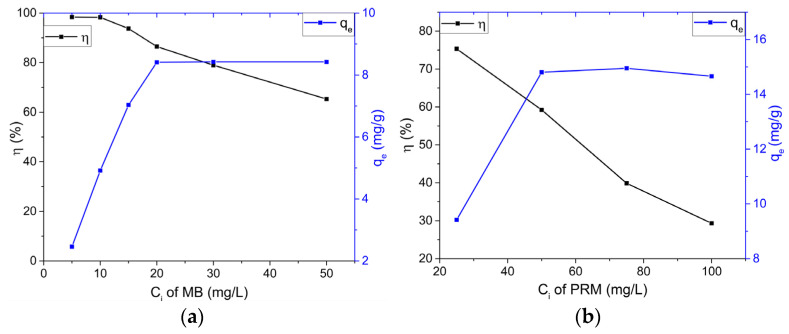
Effect of the initial pollutant concentration on the removal efficiency and maximum adsorbed amount by HCWS3; MB (**a**) and PRM (**b**). The black and blue lines represent the removal efficiency (*η*) and the adsorption capacity (*q_e_*), respectively.

In order to study the effect of the initial concentration of pollutant on the removal efficiency, the initial concentrations (*C_i_*) used were from 5 mg/L to 50 mg/L for MB and from 25 mg/L to 100 mg/L for PRM, while the other parameters were kept constant (solution volume—50 mL; adsorbent mass—0.1 g; contact time—chosen for each pollutant, according to previously obtained results: 15 min MB and 75 min PRM). Graphs of the effect of the initial pollutant concentration are shown in Figure 5a,b.

According to Figure 5a for MB, the retention efficiency of the HCWS3 material remained approximately the same for *C_i_* of 5 and 10 mg/L, after which it started to decrease gradually to a value of 68.32% at an initial concentration of 50 mg/L. The adsorption capacity of MB per gram of adsorbent increased to a maximum value of 8.412 mg/g at a *C_i_* of 20 mg/L, after which it remained constant.

In the case of PRM (Figure 5b), the decrease in retention efficiency with increasing *C_i_* was much higher, reaching values of 29.32% for *C_i_* 100 mg/L at PRM. As for MB, for this pollutant, the adsorption capacity also initially increased to reach the maximum value of 14.81 mg/g for PRM at *C_i_* of 50 mg/L.

This suggests that the adsorption is highly dependent on the initial pollutant concentration. For this reason, at a lower concentration, the ratio of the initial number of pollutant molecules to the available surface area is low and subsequently fractional adsorption becomes independent of the initial concentration. However, at a high concentration, the available adsorption centers become fewer and, therefore, the percentage removal of the pollutant depends on the initial concentration.

### 2.3. Kinetic and Adsorption Studies

**Figure 6:**
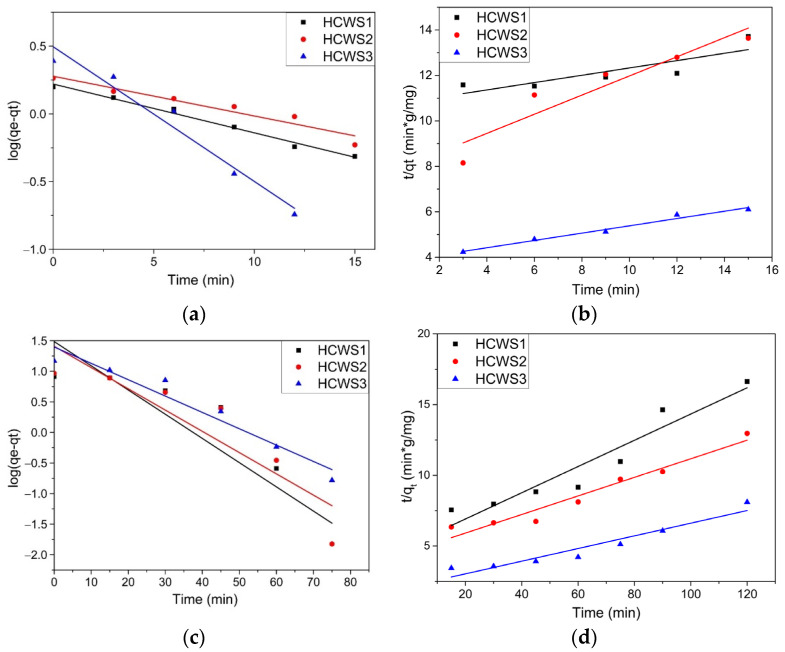
PFO kinetic model for (**a**) MB and (**c**) PRM, and PSO kinetic model for (**b**) MB and (**d**) PRM.

**Figure 7:**
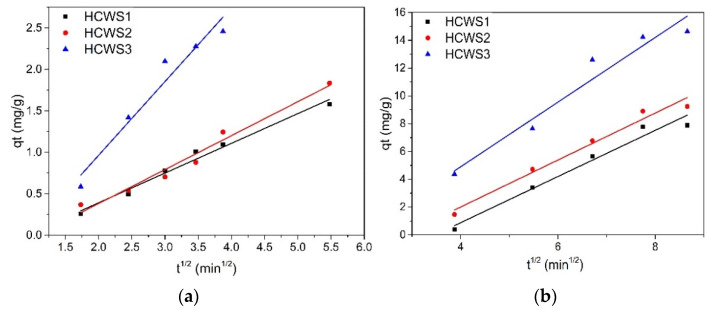
Weber–Morris intraparticle diffusion model for HCWS eco-materials, (**a**) MB; (**b**) PRM.

In order to understand how the adsorption process of MB and PRM pollutants takes place, a kinetic study was carried out for materials derived from WS biowaste by the HTC process. Linear graphs were plotted for the pseudo-first-order kinetic model (PFO) log(*q_e_ − q_t_*) versus time to determine the rate constant *k*_1_ (min^−1^) for the adsorbent eco-materials used, and linear graphs *t/q_t_* versus time to determine the pseudo-second-order kinetic model (PSO) rate constant, *k*_2_ (g/mg·min). The graphical representation for these kinetic models is shown in Figure 6a–d, and the kinetic parameters obtained, together with the value of the correlation coefficient, R^2^, are presented in Table 1. The table also contains the intraparticle diffusion model (Weber–Morris) (W–M) parameters, which are presented in Figure 7a,b.

By comparing R^2^ values between models, the PFO kinetic model best described the experimental kinetic data for MB adsorption on the surfaces of the HCWS1 and HCWS2 eco-materials (R^2^ = 0.99 and 0.93). The adsorption kinetic parameters obtained using the PFO kinetic model (theoretical *q_e_* 1.66 mg/g for HCWS1 and 1.69 mg/g for HCWS2) gave the best correlation with the experimental data, for which the *q_e_* values were 1.44 mg/g and 1.69 mg/g, respectively. In the case of MB adsorption on the surface of the HCWS3 eco-material, the experimental data are better described by PSO kinetics (R^2^ = 0.99), indicating that the adsorption process may be dominated by chemisorption for this material. Moreover, the kinetic data obtained best fit the experimental data. The kinetic study suggested rapid adsorption by a chemisorption process, and the equilibrium implied a multilayer adsorption for the HCWS3 eco-material, while, for HCWS1 and HCWS2, the process was dominated by monolayer physisorption.

For the PRM pollutant, by comparing the values of the correlation coefficients R^2^ obtained from these models, the PSO kinetic model best described the experimental kinetic data for all WS-derived materials, with R^2^ = 0.92, 0.95, and 0.992 for HCWS1, HCWS2, and HCWS3, respectively. Closer values were also observed for the experimental and theoretical determinant *q_e_* for the PSO kinetic model. The kinetic study suggested rapid adsorption through a chemisorption process, and the equilibrium implied a multilayer adsorption for all HCWS materials.

To better understand the diffusion process of pollutant molecules on the surfaces of HCWS eco-materials, the kinetic data were fitted to the Weber–Morris intraparticle diffusion model and the graph is shown in Figure 7a,b.

The linear plots *q_t_* versus *t*^1/2^ are attributed to the diffusion step through the macropores of the materials, which represent the accessible sites for the adsorption process [58]. In one study, it was indicated that the adsorption process is controlled by the intraparticle diffusion model when the graphical representation of *Q_t_* as a function of *t*^1/2^ is a straight line through the origin. If the straight line does not pass through the origin, it means that the intraparticle diffusion is not the only mechanism involved in limiting the adsorption rate [59]. It can be seen from the graphs presented in Figure 7a,b that in the case of both MB and PRM removal from all the materials, the lines did not cross the origin, which indicates that the adsorption process is a very complex one involving surface adsorption, interparticle diffusion, and intraparticle diffusion.

**Figure 8:**
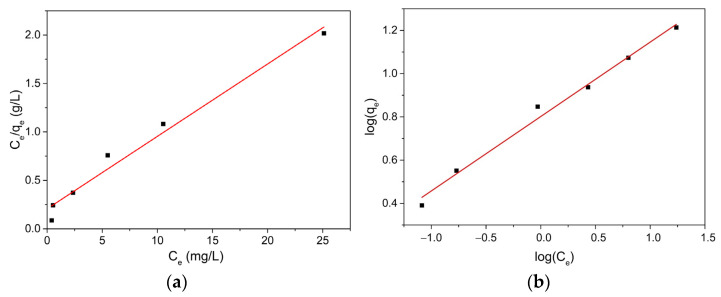

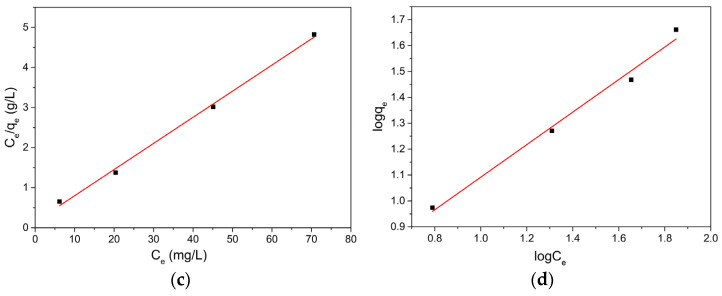
Langmuir adsorption model ((**a**) MB and (**c**) PRM) and Freundlich adsorption model ((**b**) MB and (**d**) PRM).

In order to study the distribution mechanism of adsorbed molecules between the liquid and solid phases when the adsorption process reaches a steady state, Langmuir and Freundlich adsorption isotherms, shown in Figure 8a–d, were analyzed.

The Langmuir constant correlated with adsorption capacity was determined as *Q*_0_ = 17.006 mg/g, along with the Langmuir constant *b* = 0.004 L/mg attributed to adsorbent affinity, and the correlation coefficient R^2^ = 0.9975. According to the data in the table, for the HCWS3 material, the separation factor *R_L_* was found to be between 0 and 1 for all concentrations studied, suggesting an adsorption process favorable to the Langmuir model. From the graphical representation, the *log q_e_* versus *log C_e_* were obtained: the Freundlich constant *k_F_* = 4.508 mg/g and the constant *n_F_* = 1.799, a value indicating a favorable adsorption process of MB on the material surface, and the correlation coefficient R^2^ = 0.999. 

The adsorption isotherms for PRM are shown in Figure 8c,d, and the Langmuir and Freundlich parameters are summarized in Table 2. According to the data in the table, for the HCWS3 material, it was found that the separation factor R_L_ is between 0 and 1 for all studied concentrations, suggesting an adsorption process favorable to the Langmuir model. By comparing the R^2^ of the Langmuir and Freundlich models, a better fit of both the MB and PRM adsorption processes on the surface of the HCWS3 material was found for the Freundlich model, suggesting that the material surface may exhibit heterogeneous portions with both monolayer and multilayer adsorption behavior.

## 3. Materials and Methods

### 3.1. Chemicals and Reagents

Walnuts were purchased from the local market and the shells were used as raw materials for the preparation of hydrochars. For the adsorption test, a stock solution of 1% MB (produced by Adya Green Pharma) and paracetamol tablets (500 mg PRM per tablet) purchased at a local pharmacy were used.

### 3.2. Preparation of Hydrochars

The WS were ground using a ball mill and the powder obtained was sieved using a Retsch metal sieve with a pore diameter of 250 µm. This powder was further used to obtain hydrochars. In a typical HTC process, 30 g of WS powder was mixed at a 1:10 weight ratio with distilled water; then, the mixture was loaded into a dynamic autoclave and it was sealed shut. The autoclave was heated to 220 °C with a 5 °C/min heat rate and held at this temperature for 1 h, 6 h, and 12 h, respectively. After each process, the autoclave was left to cool down at room temperature before it was opened and the mixtures (liquid phases and solid phases) were collected and separated by using a Buchner funnel equipped with a vacuum pump. The liquid phases were collected and stored for other investigations that will not be detailed in this research. The solid phases (the hydrochars—HCs) were washed with hot water several times, until the washing water remained clear, and the HC was dried in a Memmert oven at 80 °C until a constant mass was obtained. The powders were deposited in a desiccator to avoid the hydration of the eco-material with environmental water molecules. The HCs were further noted as HCWS1, HCWS2, and HCWS3 and used as adsorbent eco-materials for the removal of MB and PRM from synthetic water solutions.

### 3.3. Characterization of Hydrochars

The HCs obtained were characterized by determining the types of functional groups present on the surfaces of the eco-materials by using attenuated total reflectance Fourier transform infrared spectroscopy (ATR-FTIR) with an Interspec 200-X Spectrophotometer (Interspectrum, Tõravere, Estonia). To determine the morphology and elemental composition, scanning electron microscopy (SEM) coupled with energy-dispersive spectra (EDS) (QUANTA 450 FEG microscope, Eindhoven, The Netherlands), equipped with a field emission gun and a 1.2 nm resolution X-ray energy-dispersive spectrometer, with a resolution of 133 eV, were used.

### 3.4. Removal Efficiency and Kinetic Studies

To carry out the studies on the removal efficiency of HCWS-type adsorbent eco-materials obtained from biowaste by the HTC process, the experiments for each pollutant were carried out according to the same procedure: 0.1 g of adsorbent eco-material was brought into contact with 50 mL of pollutant solution in a 100 mL Berzelius beaker on an electric shaker equipped with a 500 rpm stirring system. Samples were taken at defined time intervals to investigate the performance of the eco-materials obtained, by determining the remaining concentrations in the solution after each determined time interval using an Orion™ AquaMate 8000 UV–VIS Spectrophotometer (Thermo Fisher Scientific, Madison, WI, USA). All the results obtained represent the average of the 3 experiments carried out, in order to demonstrate the repeatability of the procedure.

The removal efficiencies (*η*) and adsorption capacities (*q_e_*) of the eco-materials were calculated according to the following equations:

(1) $$\eta =\frac{C_{0}-C_{t}}{C_{0}}\times 100\;(\%)$$

(2) $$q_{e}=\frac{C_{0}-C_{t}}{m_{ads}}\times V\;(\text{mg/L})$$

where *η*—removal efficiency (%); *C*_0_—initial concentration of the pollutant solution (mg/L); *C_t_*—concentration of the pollutant solution at time *t* (mg/L); *q_e_*—adsorption capacity of the eco-material (mg/g); *m_ads_*—amount of adsorbent eco-material used in the experiments (g); *V*—the volume of the solution placed in contact with the adsorbent (L).

Pseudo-first-order (PFO) and pseudo-second-order (PSO) kinetic models and the Weber–Morris (W–M) model were used to perform the adsorption kinetic studies and to investigate the intraparticle diffusion.

The PFO kinetic model describes the relationship between the adsorbent centers of adsorbent and adsorbed substances [37], and the equation in its linear form is given by the following formula:

(3) $$log(q_{e}-q_{t})=logq_{e}-\frac{t}{2.303}k_{1}\;$$

where *q_e_* and *q_t_* are the amounts of pollutant adsorbed at equilibrium and at the time of sampling (mg/g), respectively, and *k*_1_ is the adsorption rate constant (min^−1^). The log(*q_e_* − *q_t_*) versus time line graph was used to determine the rate constant *k*_1_ for the adsorbent eco-materials used.

The PSO kinetic model focuses more on the chemisorption process and the dependence of adsorption capacity on time [60].

(4) $$\;\frac{t}{q_{t}}=\frac{1}{q_{e}^{2}\times k_{2}}+\frac{t}{q_{e}}\;$$

The line graph of *t/q_t_* versus time was plotted to determine the PSO rate constant and *k*_2_ (g/mg·min), according to Equation (4).

Weber–Morris is a suitable model when the diffusion of the pollutant on the internal surface or through pores inside the absorbent is the rate-limiting step. The following equation shows the Weber–Morris model:

(5) $$q_{t}=k_{3}\times t^{\frac{1}{2}}+C\;$$

where *k_3_* (mg/g·min^1/2^) is the intraparticle diffusion rate constant, and *C* is a constant proportional to the boundary layer thickness (mg/g), which can be determined by plotting the line graph of *q_t_* versus *t*^1/2^.

To further understand the distribution mechanism of adsorbed molecules between liquid and solid phases when the adsorption process reaches equilibrium, Langmuir and Freundlich adsorption isotherms were analyzed. The Langmuir model describes monolayer adsorption on the adsorption centers of adsorbent eco-materials obtained through the HTC process, and the linear form of the Langmuir isotherm is expressed as follows:

(6) $$\;\frac{C_{e}}{q_{e}}=\frac{1}{K_{L}\times Q_{0}}+\frac{C_{e}}{Q_{0}}\;$$

where *C_e_* (mg/L) is the concentration at equilibrium, *q_e_* is the amount of pollutant adsorbed, and the *Q*_0_ constant represents the adsorption capacity (mg/g) (fraction of active sites). *K_L_* (mg^−1^) is the Langmuir constant, which indicates the degree of interaction between the adsorbate and surface.

The Freundlich model describes the non-ideal and reversible adsorption processes occurring on heterogeneous surfaces and active centers of different energies, based on multilayer adsorption and equilibrium [61]. The linear form of the Freundlich isotherm model is represented as follows:

(7) $$log_{10}q_{e}=log_{10}K_{F}+\frac{1}{n_{F}}log_{10}C_{e}$$

where *C_e_*—pollutant concentration at equilibrium (mg/L); *q_e_*—the amount of pollutant adsorbed per unit adsorbent mass (mg/g); *K_F_* is the Freundlich isotherm constant (mg/g) and *n_F_* is the adsorption intensity (binding energy). The latter gives an indication of how favorable the adsorption process is, and when >1, the pollutant is favorably adsorbed on the adsorbent.

## 4. Conclusions

The three eco-materials (HCWS1, HCWS2, and HCWS3) obtained from walnut shell biowaste as a carbon source by the HTC process at different residence times were tested as potential adsorbents for two different emerging pharmaceutical pollutants: methylene blue and paracetamol. Firstly, these eco-materials were characterized in terms of the characteristic functional groups responsible for the adsorption process by ATR-FTIR analysis, and morphological features were evaluated using SEM-EDS analysis. Studies were carried out to optimize both the preparation process of the eco-materials and the adsorption and retention process of the pollutants from aqueous solutions. Factors such as contact time, amount of adsorbent used, and initial pollutant concentration were investigated.

The best results were obtained for the HCWS3 material, with a removal efficiency of 99.3% for MB and 60% for PRM. HCWS3 presented a higher content of functional groups (–OH, –COOH) present on its surface, as well as a higher content of C and O, compared to the raw materials and eco-materials prepared at 1 h and 6 h, respectively.

From the kinetic studies and Langmuir and Freundlich adsorption isotherms, it was observed that, for the HCWS3 material, rapid adsorption by a chemisorption process takes place in the case of both pollutants studied, and the equilibrium implies a multilayer adsorption. It was also concluded that a better fit of both the MB and PRM adsorption processes on the surface of the HCWS3 material was found for the Freundlich model, suggesting that the material’s surface may exhibit heterogeneous portions with both monolayer and multilayer adsorption behavior.

From all the tests carried out, it was found that the materials showed good performance for the retention of the studied pollutants, but they can be further improved by chemical activation. As a next step, studies regarding surface chemistry linked by an adsorption mechanism will be developed in order to select the proper activation method for enhancing the adsorption properties.

## Figures and Tables

**Table 1:** Kinetic parameters for MB and PRM retention while using the WS-derived HCs.

		MB			PRM		
	Parameters	HCWS1	HCWS2	HCWS3	HCWS1	HCWS2	HCWS3
**PFO**	R^2^	0.99	0.93	0.96	0.781	0.823	0.942
	*q_t_* (mg/g)	1.66	1.69	3.13	30.619	25.585	24.945
	*q_e_* (mg/g)	1.44	1.71	2.26	8.2	9.255	14.807
	*k*_1_ (min^−1^)	0.08	0.07	0.23	0.089	0.079	0.061
**PSO**	R^2^	0.93	0.88	0.99	0.92	0.95	0.992
	*q_t_* (mg/g)	6.21	2.38	2.25	10.869	15.384	14.727
	*q_e_* (mg/g)	1.44	1.71	2.26	8.2	9.255	14.807
	*k*_2_ (g/mg × min)	10.72	7.76	3.78	5.056	4.603	2.138
	R^2^	0.98	0.97	0.95	0.98	0.97	0.95
**W–M**	*C* (mg/g)	0.35	0.41	0.89	5.76	4.75	4.40
	*k*_3_ (mg/g × min^1/2^)	0.08	0.07	0.23	1.66	1.69	2.32

**Table 2:** Langmuir and Freundlich parameters for MB and PRM adsorption while using the HCWS3 adsorbent.

	Parameters	MB	PRM
Langmuir	R^2^	0.975	0.997
	*Q*_0_ (mg/g)	17.006	15.384
	*b*	0.004	0.009
	*R_L_*	0.806–0.976	0.513–0.808
Freundlich	R^2^	0.999	0.999
	*K_F_* (mg^−1^)	4.508	2.904
	*n_F_*	1.779	1.592

## Data Availability

Not applicable.
